# IL7Rα Expression and Upregulation by IFNβ in Dendritic Cell Subsets Is Haplotype-Dependent

**DOI:** 10.1371/journal.pone.0077508

**Published:** 2013-10-16

**Authors:** Fiona C. McKay, Edwin Hoe, Grant Parnell, Prudence Gatt, Stephen D. Schibeci, Graeme J. Stewart, David R. Booth

**Affiliations:** 1 Institute for Immunology and Allergy Research, Westmead Millennium Institute, Sydney, New South Whales, Australia; 2 Department of Medicine, University of Sydney, Sydney, New South Whales, Australia; University Hospital Basel, Switzerland

## Abstract

The IL7Rα gene is unequivocally associated with susceptibility to multiple sclerosis (MS). Haplotype 2 (Hap 2) confers protection from MS, and T cells and dendritic cells (DCs) of Hap 2 exhibit reduced splicing of exon 6, resulting in production of relatively less soluble receptor, and potentially more response to ligand. We have previously shown in CD4 T cells that IL7Rα haplotypes 1 and 2, but not 4, respond to interferon beta (IFNβ), the most commonly used immunomodulatory drug in MS, and that haplotype 4 (Hap 4) homozygotes have the highest risk of developing MS. We now show that IL7R expression increases in myeloid cells in response to IFNβ, but that the response is haplotype-dependent, with cells from homozygotes for Hap 4 again showing no response. This was shown using freshly derived monocytes, *in vitro* cultured immature and mature monocyte-derived dendritic cells, and by comparing homozygotes for the common haplotypes, and relative expression of alleles in heterozygotes (Hap 4 vs not Hap 4). As for T cells, in all myeloid cell subsets examined, Hap 2 homozygotes showed a trend for reduced splicing of exon 6 compared to the other haplotypes, significantly so in most conditions. These data are consistent with increased signaling being protective from MS, constitutively and in response to IFNβ. We also demonstrate significant regulation of immune response, chemokine activity and cytokine biosynthesis pathways by IL7Rα signaling in IFNβ -treated myeloid subsets. IFNβ-responsive genes are over-represented amongst genes associated with MS susceptibility. IL7Rα haplotype may contribute to MS susceptibility through reduced capacity for IL7Rα signalling in myeloid cells, especially in the presence of IFNβ, and is currently under investigation as a predictor of therapeutic response.

## Introduction

Multiple Sclerosis (MS) is the most common chronic neurological disease in young adults. Both genetic and environmental factors are implicated and interact in mediating pathogenesis [1]–[6]. The common polymorphisms mediating susceptibility to MS reside predominantly within, and neighboring, the genes affecting immune function, including the interferon response genes [2], [7]. The challenge of the next phase of genetic research in MS is to identify the functional impact of these newly identified variants, possibly including a differential response to environmental susceptibility factors such as reduced vitamin D and viral infection [3], [5].

We and others found an association of the interleukin-7 receptor α chain (IL7Rα) with MS [2], [8]–[10] and have identified key functional differences between IL7Rα haplotypes that are likely to affect MS susceptibility [10]–[13]. IL7Rα is a subunit of receptors for IL-7 and thymic stromal lymphopoietin (TSLP) and is expressed on T cells, dendritic cells (DCs) and other myeloid cells [14]. IL-7 is a critical and nonredundant cytokine mediating survival and differentiation of T cells and their progenitors [15], [16]; while TSLP mediates T cell proliferation and the development of Th2 and regulatory T cells (Tregs) primarily through effects on dendritic cells [17]–[22]. IL7Rα has four major haplotypes [23]. Haplotype 2 (Hap 2) is protective against MS and cells exhibit reduced splicing of exon 6 from the transcript, producing less of a soluble isoform (sIL7Rα) [10], [12]. However, we found that haplotype differences in splicing are greatly magnified in DCs compared to CD4 T cell subsets and PBMCs [12]. Thus, in the present study, we have examined in detail the effect of IL7Rα haplotype on myeloid cell subsets as potential drivers of the associated MS susceptible/protective phenotypes.

IL7Rα signaling exerts powerful effects on myeloid cell function, dependent on the microenvironment and on differentiation status. In monocytes, IL-7 induces inflammatory chemokines, IL-8 and MIP1β, and both TSLP and IL-7 induce the expression of the Th2-attracting chemokines, CCL17 and CCL22, [18]. TSLP skews peripheral DCs to induce an inflammatory Th2 phenotype in T cells however, in the tolerising environment of the gut, TSLP acts on intestinal DCs to induce a non-inflammatory Th2 phenotype characterized by production of the anti-inflammatory cytokine IL-10, and has been suggested to be involved in promoting tolerance to microflora and food antigens [24], [25]. Importantly, TSLP acts on thymic DCs to stimulate the production of Tregs from CD4+CD25- single-positive thymocytes [21], [26].

In MS, a defect in thymic output of natural Tregs has been observed, and correlated with reduced suppressive capacity [27]–[29]. In addition, a defect in inducible Tregs in MS has been suggested by impaired IL-10 production by Tr1 cells induced *in vitro* by CD46 costimulation [30], [31]. Th2 markers are reduced in the peripheral blood of untreated MS patients [32], and immunomodulatory therapies are associated with skewing towards a Th2 profile [33]. We hypothesise that IL7Rα haplotype may influence susceptibility to MS by altering IL7Rα pathways in myeloid cells, such as those controlling thymic Treg output, and recruitment and differentiation of Th2/Tregs in the periphery.

While Hap 2 is protective against MS, Hap 4 homozygotes carry the highest risk of MS (odds ratio 1.35; p = 0.0011) [11]. We hypothesized that there are mechanisms in addition to altered exon 6 splicing by which IL7Rα haplotype affects MS susceptibility. In support of this, we previously demonstrated that IL7Rα is upregulated by IFNβ in CD4 T cells, except in Hap 4 homozygotes [11]. IFNβ exerts complex effects on innate and adaptive immunity and plays an important role in immune defense against viral infection. Both pro- and anti-inflammatory actions have been described [34], [35]. Two recent reports have demonstrated that the anti-inflammatory effect of endogenous IFNα/β is mediated, not through direct effects on T cells, but via the IFN/β receptor on myeloid cells in the murine model of MS, experimental autoimmune encephalomyelitis [36], [37]. IFNβ is also an important first line immunomodulatory therapeutic for MS. IFNβ therapy increases thymic output of Tregs [29] and skews the T effector profile to Th2 [33], [38], mediated, at least in part, by effects on monocytes and DCs [39]. These data support a model in which IFNβ upregulation of IL7Rα enhances TSLP/IL7 signaling in myeloid cells.

To investigate potential pathways affecting MS susceptibility, we determined the effects of haplotype on IL7Rα expression and splicing in myeloid cells *ex vivo*, and *in vitro*. We found effects of Hap 2 and Hap 4 on IL7Rα expression in all myeloid subsets examined and demonstrated distinct gene expression signatures in response to IL7Rα signalling depending on ligand, differentiation status of the responder cell and IFNβstimulation. The results suggest that the MS-protective haplotype is associated with increased IL7Rα expression, especially of the membrane-bound isoform, in monocytes and dendritic cells (Hap 2), that the cytokine IFNβ increases IL7Rα expression in these cells, and that absence of this IFNβ-responsiveness in dendritic cells is associated with the highest MS risk (Hap 4 homozygotes). These studies highlight the complex effects of IL7Rα haplotype in monocytes and DC subsets, and expand our understanding of pathways controlled by protective and susceptibility variants in MS.

## Results

We had previously shown that functional differences between the MS-protective and -susceptible IL7Rα haplotypes are greatly magnified in monocyte-derived DCs compared to T cells [12]. In addition, the genotype with the highest risk of MS is unresponsive to IFNβ in T cells, but this response has not been characterized in DCs or other myeloid cells. In this study we examine IL7Rα expression and function in myeloid cell subsets in detail, with the aim of identifying contexts potentially relevant to MS pathogenesis and response to IFNβ therapy.

We first compared expression of IL7Rα and a major splice form in monocytes, *ex vivo* DC subsets (myeloid DCs and plasmacytoid DCs) and *in vitro*-generated DCs at two stages of differentiation (immature and maturing). We also determined whether expression and splicing change in response to IFNβ stimulation. Secondly, we determined whether expression, splicing and response to IFNβ differ between the IL-7R haplotypes at various stages of differentiation. Thirdly, to test the possible functional relevance of haplotype differences in IFNβ response, we characterized gene profiles and immune pathways regulated by IL-7 and TSLP in IFNβ-treated myeloid subsets. In addition, we induced a tolerogenic phenotype in dendritic cells using IFNβ, and investigated whether TSLP in this context induces inflammatory or anti-inflammatory effects on T cell proliferation.

### IL7Rα mRNA Expression and Response to IFNβ in Monocytes and DC Subsets

**Figure 1:**
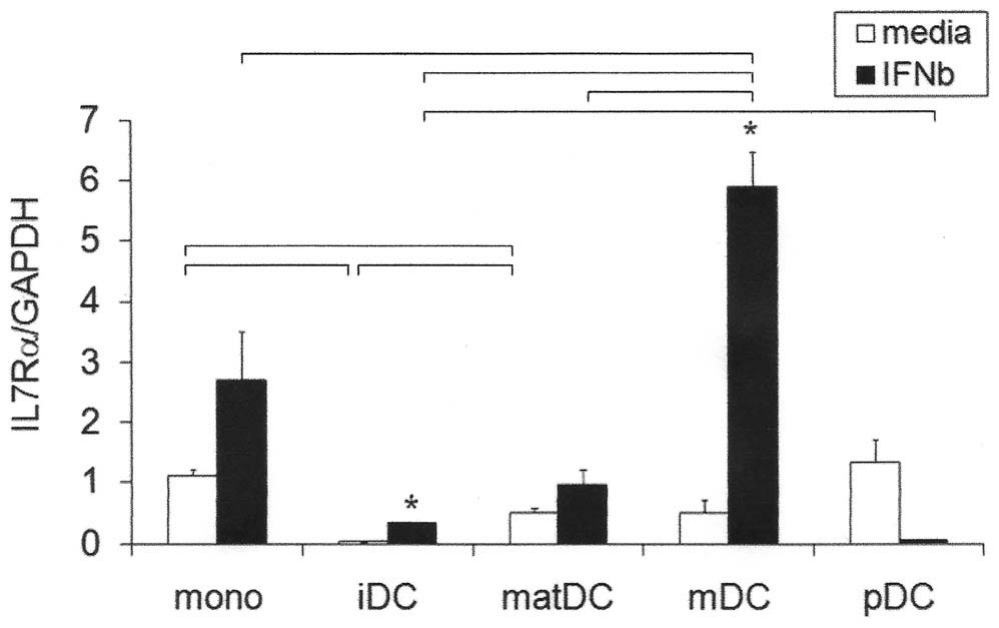
Constitutive IL7Rα gene expression and upregulation in response to IFNβin myeloid cell subsets. Freshly purified monocytes (mono), myeloid dendritic cells (mDC) and plasmacytoid dendritic cells (pDC) and *in vitro* cultured immature (IL-4, GM.CSF; iDC) and maturing monocyte-derived dendritic cells (IL-4, GM.CSF, LPS; matDC) from healthy control heterozygous carriers of Hap 4 (n = 3, each in triplicate; except for pDCs, each in duplicate) were incubated +/− IFNβ (1000 IU/ml; with the exception of pDCs at 2000 IU/ml) for 24 h. IL7Rα was measured by RTPCR relative to GAPDH; mean +/− SEM is shown; *significantly different from media condition by paired t test (iDC, p = 0.006; mDC, p = 0.005); bars represent significant differences between subsets under the same condition by Student's t test (p<0.05).

Total IL7Rα relative expression and response to IFNβ were measured in *ex vivo* and *in vitro* generated myeloid cell subsets from healthy controls (Figure 1; Hap2/Hap4 heterozygotes; n = 3). In the absence of IFNβ, there were significant differences between the subsets in IL7Rα expression (monocytes > maturing DC > immature DC; all p<0.05). In the presence of IFNβ, myeloid DCs expressed significantly higher levels of IL7Rα than all other subsets (all p<0.05). In response to IFNβ, mean IL7Rα expression was increased in monocytes (2.5 fold, not significant (ns)), maturing DCs (1.6 fold, ns), immature DCs (12 fold; p = 0.006) and myeloid DCs (10 fold; p = 0.005). There was a trend towards downregulation of IL7Rα by IFNβ in plasmacytoid dendritic cells (pDCs) (10 fold; p = 0.071). (pDCs were conditioned with a higher IFNβ dose (2,000 IU/ml) since lower doses of IFNβ result in spontaneous apoptosis [40]). These represent larger changes in response to IFNβthan we had previously measured for CD4 T cells (1.2 fold) and PBMCs (2 fold) [11].

### Differential Expression of IL7Rα Haplotypes in Myeloid Subsets

#### Relative haplotype expression compared in homozygous cells

**Figure 2:**
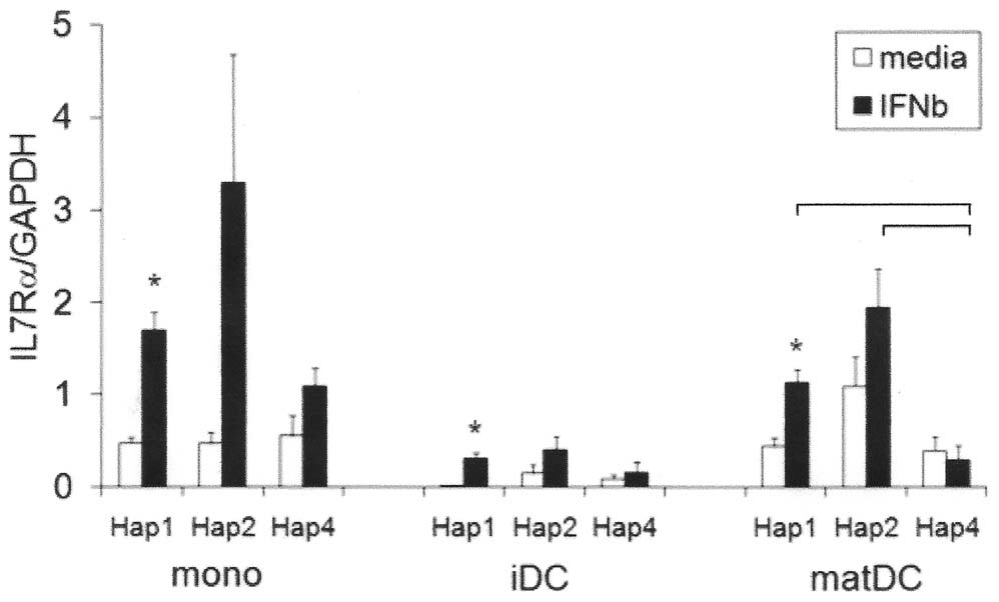
IL7Rα Hap 4 is not upregulated on stimulation with IFNβ in myeloid cells of homozygotes. Freshly purified monocytes (mono), *in vitro* cultured immature (IL-4, GM.CSF; iDC) and maturing monocyte-derived dendritic cells (IL-4, GM.CSF, LPS; matDC) from healthy control homozygotes of Hap 1, Hap 2 and Hap 4 were incubated +/− IFNβ (1000 IU/ml) for 24 h (n = 3, each in triplicate). IL7Rα was measured by RTPCR relative to GAPDH; mean +/− SEM is shown; *significantly different from media condition by paired t test (Hap 1 mono, p = 0.027; iDC, p = 0.046; matDC, p = 0.026); bars represent significant differences between haplotypes in the same subset under the same condition by Student's t test (p<0.05).

Of the 3 haplotypes, IL7Rα Hap 2 was consistently expressed at the highest level in all IFNβ-treated myeloid subsets examined (1.3–6.6 fold higher), as well as in untreated dendritic cell subsets (1.7–6.5 fold higher) (mean values, ns; Figure 2). Similar to our previous findings in CD4 T cells, IL7Rα Hap 4 was not upregulated in response to IFNβ in any of the subsets (Figure 2). In contrast, Hap 1 was upregulated in all subsets in response to IFNβ (mono, p = 0.027; iDC, p = 0.046; matDC, p = 0.026), and mean Hap 2 IL7Rα levels were increased in all subsets (ns). As a result, Hap 4 was consistently expressed at the lowest level of all the haplotypes in all IFNβ-treated cells (means), significantly lower than Hap 1 (p = 0.021) and Hap 2 (p = 0.046) in maturing DCs.

#### Relative haplotype expression measured in heterozygous cells

**Figure 3:**
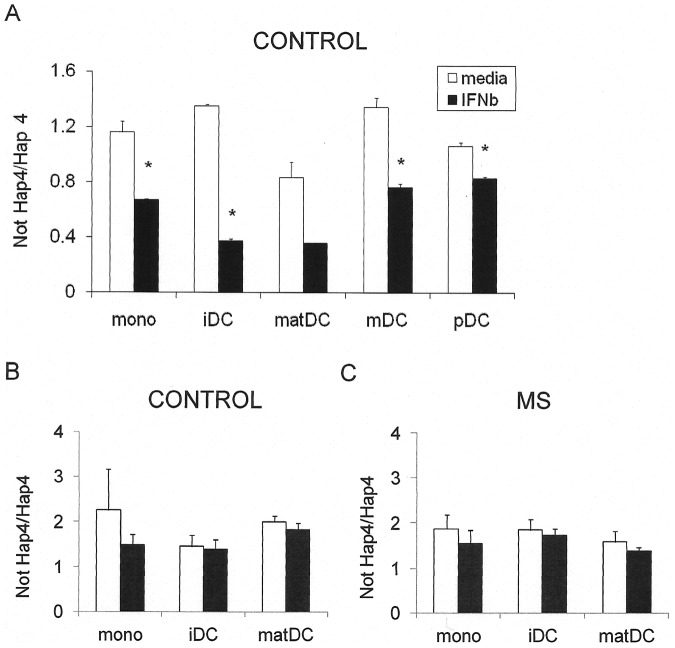
IL7Rα Hap 4 is less responsive to IFNβ than Hap 1 or Hap 2 in myeloid cells of heterozygous Hap 4 carriers. (A) Freshly purified monocytes (mono), myeloid dendritic cells (mDC) and plasmacytoid dendritic cells (pDC) and in vitro cultured immature (IL-4, GM.CSF; iDC) or maturing monocyte-derived dendritic cells (IL-4, GM.CSF, LPS; matDC) from healthy control heterozygous carriers of Hap 4 (n = 3, each in triplicate, except for pDCs, each in duplicate) were incubated +/− IFNβ (1000 IU/ml; with the exception of pDCs at 2000 IU/ml) for 24 h. Subjects were heterozygous carriers of Hap 4, bearing either Hap 1 or Hap 2 as the other allele. Expression of each haplotype was measured using tagging SNPs as previously described [13] and is presented as a ratio of expression of Hap 4/Not Hap 4 (i.e. Hap 4/Hap 1 or Hap 4/Hap 2) for each individual. In response to IFNβ, Hap 4 was expressed at a relatively lower level in all subsets than in the absence of IFNβ. Mean +/− SEM is shown; *significantly different from media condition by paired t test (mono, p = 0.025; iDC, p = 0.0003; mDC, p = 0.012; pDC, p = 0.022; mat DC showed a trend, p = 0.052). Monocytes from healthy control (n = 5) (B) or MS (n = 5) (C) heterozygous carriers of Hap 4 were purified from thawed cryopreserved PBMCs, differentiated into iDC or mDC and treated with IFNβas above. No differences in haplotype response were seen between MS and controls. A significant proportion of the combined cohort from frozen cells (n = 10; healthy control + MS) decreased relative expression of Hap4 in response to IFNβwhen all cell subsets were included in the analysis (p = 0.043, sign test); this did not reach significance for individual groups or cell subsets.

Relative expression of haplotypes can be measured under identical cellular conditions by quantitating the relative abundance of message containing haplotype-tagging SNPs in heterozygous cells. In response to IFNβ, IL7Rα Hap 4 transcript (rs3194051 ‘G’) was expressed at a relatively lower level compared to Hap 1 or Hap 2 (both rs3194051 ‘A’) than in the absence of IFNβ. Specifically, this was evident in monocytes (p = 0.025), immature DCs (p = 0.0003) and myeloid DCs (p = 0.012), with a trend in maturing DCs (p = 0.052) (Figure 3A). These results are consistent with relative expression data in homozygotes (Figure 2), where the Hap 4 alleles are less responsive to IFNβ than Hap 1 and Hap 2. However in plasmacytoid DCs, which downregulate IL7Rα on IFNβ stimulation, the response (reduction) to IFNβ is greater in Hap 4 (p = 0.022). Relative expression of Hap 4 was measured in monocytes, immature DCs and maturing DCs purified from previously frozen PBMCs from a second cohort of healthy controls (Figure 3B) and from MS patients (Figure 3C). A significant proportion of the combined cohort (n = 10; healthy control + MS) decreased relative expression of Hap4 in response to IFNβ when the 3 cell subsets (mono, iDC, mDC) were included in the analysis (p = 0.043, sign test); this did not reach significance for individual groups or cell subsets, The trend was the same in MS and controls.

#### Haplotype differences in IL7Rα splicing and isoform expression

**Figure 4:**
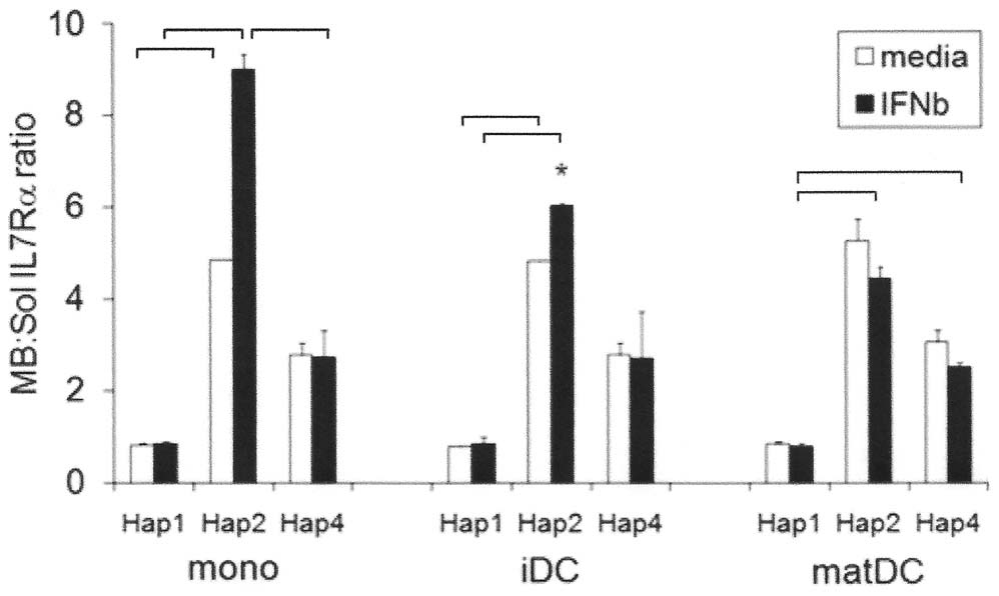
Haplotype effects on constitutive and IFNβ-stimulated splicing of IL7Rα. Freshly purified monocytes (mono), *in vitro* cultured immature (IL-4, GM.CSF; iDC) and maturing monocyte-derived dendritic cells (IL-4, GM.CSF, LPS; matDC) from healthy control homozygotes of Hap 1 (n = 3), Hap 2 (n = 2) and Hap 4 (n = 2) were incubated +/− IFNβ (1000 IU/ml) for 24 h with triplicates for each individual. Relative expression of membrane-bound (full-length) and ex 6 soluble (sol) IL7Rα isoform was measured as previously described [13] and expressed as a ratio. IFNβ increases the ratio of membrane-bound to ex 6 solIL7Rα receptor isoform expressed in myeloid cells of Hap 2 (Hap 2 iDC, p = 0.026; Hap 2 mono showed a trend, p = 0.056). Mean +/− SEM is shown; *significantly different from media condition; bars represent significant differences between haplotypes in the same subset under the same condition by Student's t test (p<0.05).

We had previously shown lower production of a soluble isoform, capable of inhibiting IL7Rα signaling in T cells and DCs of Hap 2 compared to the other haplotypes [12]. Here we determined the relative expression of this soluble isoform of IL7Rα in myeloid subsets, and whether this changes in response to IFNβ (Figure 4). The ratio of transcript encoding membrane-bound isoform to this soluble isoform (MB:Sol), generated by splicing out of exon 6, was determined as previously described [13] (Figure 4). Hap 2 expressed a higher ratio of membrane-bound to soluble isoform than Hap 1 in all IFNβ-treated myeloid subsets examined, and also in monocytes and immature DCs in the absence of IFNβ(Figure 4). The mean ratio for Hap 4 was intermediate in all subsets and conditions (significantly lower than Hap 2 in IFNβ-treated monocytes, and significantly higher than Hap 1 in IFNβ-treated maturing DCs). IFNβ treatment increased the relative expression of the membrane-bound isoform only in Hap 2 (significant in immature DCs) (Figure 4). This is consistent with our finding that Hap 4 heterozygous cells showed no change in relative expression of the membrane-bound and soluble isoforms in response to IFNβ (Figure S1), including a second smaller and less abundant soluble isoform generated by splicing out of exon 5 and 6 [41] either in healthy controls or MS. We also found no difference between healthy controls and MS in relative expression of membrane-bound and soluble isoforms, either in the presence or absence of IFNβ.

### Functional Significance of IL7Rα Signaling in IFNβ-treated DCs

#### Gene expression

Having demonstrated that the protective haplotype is most highly expressed, and the risk haplotype is expressed at lowest levels in IFNβ-treated myeloid subsets; we determined the immune genes and pathways regulated by IL7Rα signaling and thus potentially affecting MS risk in these subsets.

**Figure 5:**
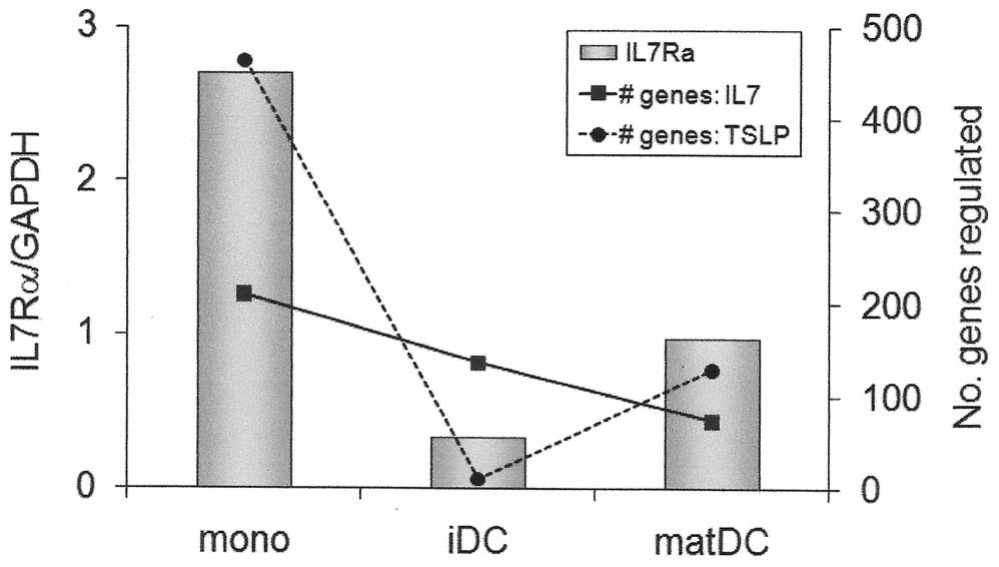
Magnitude of response to IL-7 or TSLP is dependent on IL7Rα expression in IFNβ-treated myeloid cell subsets. Freshly purified monocytes (mono), *in vitro* cultured immature (IL-4, GM.CSF; iDC) and maturing monocyte-derived dendritic cells (IL-4, GM.CSF, LPS; matDC) from a heterozygous Hap2/Hap4 individual were incubated with IFNβ (1000 IU/ml) for 24 h. Bars represent the level of expression of IL7Rα after 24 h of IFNβ stimulation (IL7Rα). IL-7 or TSLP (10 ng/ml) were added at this point, and gene expression measured 24 h later. Lines represent the number of genes up- or down-regulated at least 1.5-fold by IL-7 (# genes: IL7) or TSLP (# genes: TSLP) as assessed by microarray. (These data also presented as individual numbers of up- and down-regulated genes in Table 1).

IL-7 and TSLP significantly altered gene expression patterns in IFNβ-treated monocytes, immature DCs and maturing DCs (Table 1). Of the differentially regulated genes, IL-7 upregulated a larger proportion in monocytes and immature DCs, but downregulated a larger proportion in maturing DCs. In contrast, TSLP up- and down-regulated approximately equal proportions of genes in each subset. Importantly, the number of genes differentially regulated by either ligand was related to the level of IL7Rα expression, particularly so for TSLP (Figure 5). This underscores the functional significance of differences in IL7R expression, suggesting that lower-expressing haplotypes and lack of response to IFNβ may confer reduced response to IL-7 and TSLP.

**Table 1:** Gene expression in response to IL7Rα signaling in IFNβ-treated DC subsets^1^.

	IL7	TSLP
**Monocytes**	**All genes** 2: 143 ↑, 67 ↓	**All genes**: 215 ↑, 248 ↓
	**Pathways** 3: none identified	**Pathways**: ↓ immune response^9^
**Immature DCs**	**All genes**: 79 ↑, 57 ↓	**All genes**: 5 ↑, 5 ↓
	**Pathways**: ↑ chemokine activity^4^ ↑ immune system response^5^	**Pathways**: none identified
**Maturing DCs**	**All genes**: 16 ↑, 57 ↓	**All genes**: 68 ↑, 61 ↓
	**Pathways**: ↑ up-regulation of cytokine biosynthetic process^6^ ↓ chemokine activity^7^ ↓ immune system response^8^	**Pathways**: none identified

1 freshly purified monocytes or monocyte-derived DCs were preincubated for 48 h with IFNβ (1000 IU/ml; +LPS for maturing) before 24 h stimulation with IL7 or TSLP (10 ng/ml) and gene expression measured by microarray.

2 total number of genes upregulated (↑) or downregulated (↓) at least 1.5-fold by IL-7 or TSLP. This data is also presented as a sum of total genes regulated in Figure 5.

3 gene ontology (GO; http://www.geneontology.org) pathways identified in lists of genes upregulated or downregulated at least 1.5-fold by IL7 or TSLP. Pathway number, names and numbers of differentially regulated members of the pathway and p values, comparing pathway representation in up- or down-regulated gene lists compared to all expressed genes, are given for each condition below.

4 GO:0008009; ***LOC728835***, *CXCL10*, *CCL4L1*, *CCL22*, *CCL17*, ***IL8***; (n = 6; p<6×10^−7^; genes overlapping with list g shown in bold).

5 GO:0002376; *NF*κ*BIA*, *RGS1*, *CCL4L1*, *MMP9*, *KLF6*, *CCL17*, *IL8*, *LOC728835*, *CXCL10*, *FCER1A*, *CCL22*, *CCL4L2*, *CST7*, *CTLA4*; (n = 14; p<2×10^−3^; genes overlapping with list h underlined).

6 GO:0042108; *CD86*, *FCER1A*; (n = 2; p<0.020).

7 GO:0008009; *CCL2*, *CCL4*, *CCL5*, *CCL18*, *CCL13*, *CXCL16*, ***IL8***, ***LOC728835***, *CCL4L2*, *CXCL1*, *CCL8*; (n = 11; p<2×10^−16^; genes overlapping with list d shown in bold).

8 GO:0002376; *CCL2*, *CCL4L1*, *MMP9*, *CXCL16*, *IL8*, *LOC728835*, *LILRA3*, *TIMP1*, *IFITM2*, *CCL5*, *CCL18*, *CCL13*, *HLA-DRA*, *C1QB*, *HLA-DPA1*, *C1QC*, *CCL4L2*, *HLA-DRB6*, *CXCL1*, *CCL8*; (n = 20; p<4×10^−14^; genes overlapping with list e underlined).

9 GO:0006955; *OAS2*, *RGS1*, *IL1RN*, *LAG3*, *CD86*, *IGLL1*, *CD74*, *C1QB*, *IGSF6*, *C1QC*, *HLA-DQA1*, *CXCR4*, *CLEC7A*, *LOC554223*, *HLA-C*, *RBM4*; (n = 16; p<0.048).

IL-7 upregulated chemokine activity and immune system response pathways in IFNβ-treated immature DC, including chemokines of the CXC (*IL8*) and C-C chemokine-ligand/-like families (*CCL4L1, CCL4L2*) and a matrix metalloproteinase (*MMP9*), but downregulated these pathways (and these four pathway members) in IFNβ-treated mature DCs (Table 1). However overall, individual pathway members upregulated by IL-7 in these immature DCs mostly did not overlap with members downregulated by IL-7 in maturing DCs. Of interest, IL-7 upregulated the chemokine genes *CCL17* and *CCL22* in IFNβ-treated immature DCs, and downregulated class II MHC genes in IFNβ-treated maturing DCs (*HLA-DRA, HLA-DPA1, HLA-DRB6*). IL-7 also upregulated the cytokine biosynthetic process pathway in IFNβ-treated maturing DCs. While no immune pathways were identified as regulated by IL-7 in IFNβ-treated monocytes, IL-7 upregulated *INDO* (encoding indoleamine-2,3-dioxygenase) and *CCR2* and similar to TSLP, downregulated *C1QB* and *C1QC*.

TSLP downregulated the immune response pathway in IFNβ-treated monocytes, including a costimulatory molecule (*CD86*), antigen presentation (*CD74, HLADQA1, HLA-C*) complement (*C1QB, C1QC*) and chemokine receptor (*CXCR4*) genes; but upregulated *IL8*, *CCL2*, and *CCL22*. No immune pathways were identified as regulated by TSLP in IFNβ-treated DC subsets, although in these maturing DCs, TSLP downregulated antigen processing molecules (*CD74, HLADRB4*) and, similar to IL-7, downregulated *MMP9* and *CCL8*.

### Effects on T cell proliferation

TSLP can induce the generation of tolerogenic DCs in the thymus and the periphery, in certain circumstances, but induces an inflammatory phenotype in others. We investigated the function of TSLP in a tolerogenic environment induced by IFNβ,to determine whetherits actions are anti-inflammatory or proinflammatory in this context. We tested (1) if IFNβ treatment induced a tolerogenic DC profile, and (2) if TSLP signaling in this context would stimulate or inhibit T cell proliferation in subsequent co-culture.

**Figure 6:**
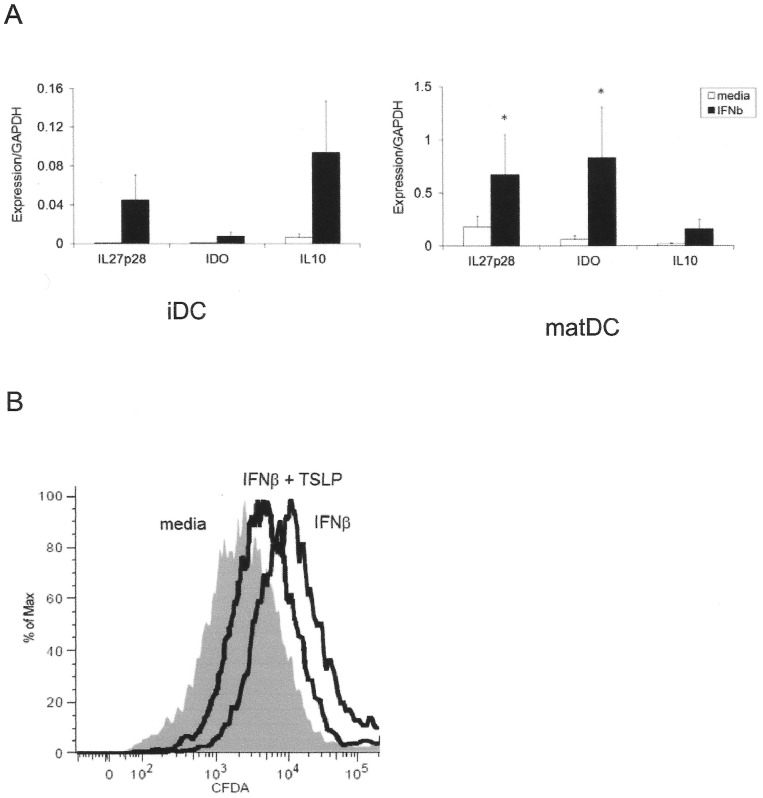
TSLP treatment of IFNβ-stimulated dendritic cells induces T cell proliferation in co-culture (A) IFNβ induces expression of tolerogenic genes in dendritic cells. Expression of IL27p28, IDO and IL10 was measured in *in vitro* cultured immature (IL-4, GM.CSF; iDC) and maturing monocyte-derived dendritic cells (IL-4, GM.CSF, LPS; matDC) (each in triplicate). Expression was measured by RTPCR relative to GAPDH; mean +/− SEM is shown; *significantly different from media condition by paired t test (IL27p28, p = 0.045; IDO, p = 0.016). (B) IFNβ-treated DCs (IFNβ) reduce proliferation of CD4 T cells compared to untreated DCs (media), but subsequent DC treatment with TSLP partially reverses this effect (IFNβ + TSLP). CFDA-loaded naïve CD4 T cells and antiCD3/antiCD28 beads were added on day 8 to maturing DC culture previously conditioned +/− IFNβ (day 5) and +/− TSLP (day 7). Proliferation of CD4+ cells was assessed on day 13 by reduction in CFDA median fluorescence intensity. The results are representative of two independent experiments.

IFNβ-conditioned immature and maturing DCs upregulated expression of tolerogenic genes IL27p28, IDO and IL10 (Figure 6A) [42]–[44]. IL10 is known to inhibit Th1 and Th17 differentiation [45], IL27p28 inhibits Th17 differentiation and proliferation [46], [47] and IDO inhibits T cell proliferation by decreasing the availability of tryptophan, an essential metabolite [48]. Accordingly, IFNβ-treated DCs suppressed the proliferation of co-cultured autologous naïve CD4 T cells during TCR stimulation (Figure 6B). However, subsequent TSLP treatment of IFNβ-treated DCs partially reversed this tolerogenic effect of IFNβ, enhancing proliferation of T cells in co-culture (Figure 6B).

## Discussion

Our driving hypothesis is that IL7Rα haplotypes are associated with MS because they alter expression of the gene. Under this hypothesis, where there are no haplotype-specific effects, the cell subset/microenvironment does not contribute to the association of the genotype with MS; and conversely, where there are large haplotype effects, the cell subset/microenvironment is likely to contribute to pathogenesis. In the absence of IFNβ, large haplotype differences were found in immature and LPS-stimulated maturing DCs, with the protective haplotype having the highest expression. With IFNβ stimulation, monocytes, immature DCs and maturing DCs displayed the same trend with highest expression by the protective haplotype, and additionally, lowest expression by the highest risk haplotype. Under all conditions, in all subsets, the protective haplotype preferentially expressed the membrane-bound isoform. Using microarray analysis, we found that cellular response to IL-7 and TSLP was related to the level of IL7Rα expression in myeloid cells. Taken together, these results suggest that the MS-protective haplotype is associated with increased capacity of dendritic cells and monocytes to respond to IL-7 or TSLP in these contexts.

IL-7 is produced by stromal cells at a fixed level, and cellular competition for this limited resource is determined by the level of IL7Rα expression [16], with the soluble IL7Rα isoform able to competitively inhibit cell-surface binding [49], [50]. We had previously demonstrated that T cells and inflammatory DCs of the protective haplotype produce a lower ratio of the soluble isoform [12]; and here show that this is also the case in immature DCs and monocytes, including under IFNβ stimulation. We also demonstrate here that splicing is not different between MS and controls in Hap 4 heterozygous carriers. T cells presumably contribute substantially to haplotype differences in serum/extracellular fluid levels of sIL7Rα, by virtue of their abundance in peripheral blood. However, we hypothesise that any given concentration of sIL7Rα may have a more significant inhibitory effect on IL7Rα signaling in DCs/monocytes than in T cells, because of the lower cell-surface receptor density of the former (10–100-fold less than T cells) [12]. In addition, we found earlier that haplotype differences in splicing are magnified in DCs compared to CD4 T cell subsets and PBMCs, with almost equimolar ratios of membrane-bound and soluble receptor generated by DCs of the susceptibility haplotype in the microenvironment [12]. This may be of particular relevance where DCs themselves [51]–[53] or cells within the immediate microenvironment [54] produce IL-7 or TSLP. Overall, increased sIL7Rα generated by T cells and DCs of the susceptible haplotypes may have a greater inhibitory effect on IL7Rα signaling in DCs and monocytes.

The functional effects of IL7Rα signalling in myeloid cells are pleiotropic and microenvironment-dependent. Based on our finding that increased myeloid cell expression of IL7Rα is associated with the MS-protective haplotype, and current knowledge of IL7Rα biology in myeloid cells, we suggest that several pathways may be worthy of further investigation as potential contributors to MS susceptibility. Firstly, both IL-7 and TSLP induce expression of the Th2-attracting chemokines CCL17 and CCL22 in monocytes [18], [19]. Here we also demonstrated induction of these chemokines by IL7/TSLP in IFNβ-treated myeloid cells. The expression of these renders subsequently differentiated monocyte-derived DCs highly chemoattractive to regulatory T cells, and skews the balance of effectors to CD4 rather than CD8 [55]. Skewing of the T helper balance to Th2/Treg recruited at sites of inflammation [56]–[58] is one potential mechanism by which higher IL7Rα signaling might protect against autoimmunity.

Secondly, TSLP also acts on DCs to tolerise T cells in at least two important contexts of potential relevance to MS: the gut and the thymus. There is accumulating evidence that the relationship between host immunity and gut microbiota may influence the development of autoimmunity (reviewed in [59]). TSLP is constitutively expressed in healthy human colon and is inducible in a human enterocyte cell line upon exposure to gut commensals, acting on intestinal DCs to induce a non-inflammatory Th2 phenotype characterized by IL-10 and IL-13 production [24], [25]. Induction of a more tolerogenic gut DC phenotype [60] and increases in IL-10 and IL-13 [61] by manipulation of gut microbiota are associated with attenuation of subsequently induced experimental autoimmune encephalomyelitis. Reduced IL-17 and interferon gamma in both intestine and spinal cord in these animals suggests that the intestinal microenvironment can influence neurologic inflammation [60]. A second mechanism by which higher myeloid expression of IL7Rα could potentially confer protection from MS is via increased tolerising effects of TSLP in the gut.

Thirdly, TSLP also acts on DCs in Hassall's corpuscles of the thymus to induce Treg production from CD4+CD25- thymocytes [20], [26]. It has been suggested recently that minor perturbations in the number or function of Tregs may be permissive for the development of autoimmune disease, allowing differentiation and proliferation of low affinity self-reactive CD4 T cells that have escaped thymic deletion [62]. In MS, reduced thymic Treg production has been observed [27]–[29], while current first-line immunomodulatory therapies increase thymic Treg output [29]. Higher IL7Rα expression by the MS-protective haplotype could potentially improve thymic TSLP signaling and Treg output.

In the presence of IFNβ, IL7Rα was upregulated in all myeloid subsets examined, and expressed at the highest level in myeloid DCs (10-fold upregulated), suggesting these cells may be the most responsive to IL7Rα signaling under conditions of IFNβ stimulation. Both IL-7 and TSLP induced significant changes in expression of immune pathways in IFNβ-treated monocytes and monocyte-derived DCs, indicating that the upregulation may be of functional significance. IFNβ stimulation also unmasked a functional difference in the haplotype conferring highest MS risk: lack of upregulation and consequent lowest expression of IL7Rα in IFNβ-treated dendritic cells. IFNβ is specifically induced at sites of viral and other microbial infections [35], [63] and autoimmune inflammation [64] including the acute MS lesion [65], but is also constitutively expressed at high levels by thymic epithelial cells under non-inflammatory conditions, as shown recently using a reporter mouse model [63]. Attenuated responses of DCs, particularly myeloid DCs, to IL-7 or TSLP in the presence of IFNβ, such as during microbial infection, central nervous system inflammation or in the thymus, may contribute to increased MS risk in Hap 4 homozygotes.

Whether upregulation of IL7Rα is involved in the clinical effect of IFNβ is not yet known. IFNβ treatment increases Treg output from the thymus with concomitant improvements in Treg suppressive function [29], and skewing of the T cell phenotype to Th2 [33], [39] has been associated with a long-term favorable clinical response [38]. These effects could potentially result from enhanced TSLP signaling. We are currently investigating the effect of IL7Rα haplotype on immune and clinical response to IFNβ in MS patients. To further characterize the effects of TSLP in a tolerogenic environment induced by IFNβ, we examined whether subsequent TSLP treatment of IFNβ-stimulated DCs would exert an additive suppressive capacity on TCR-mediated T cell proliferation. TSLP partially reversed the suppressive effect of IFNβ in this context. These *in vitro* conditions might not mimic a microenvironment in which improved IL7Rα signaling reduces an autoimmune inflammatory process.

Interestingly, IL7Rα was downregulated by IFNβ in DCs of the lymphoid lineage, the plasmacytoid DCs. Plasmacytoid DC precursors are the primary producers of type I IFN in response to viruses such as HSV [66] and induction of IFNβ by viral infection has been demonstrated recently using a reporter mouse model [67]. However the amount of type I IFN produced in response to virus is tightly controlled by other cytokines, with IL-7 increasing production [66]. We hypothesise that the downregulation of IL7Rα we observed in response to IFNβ represents a negative feedback loop once IL-7-primed pDCs have secreted type I IFN.

The immunomodulatory effects of IFNβ are diverse. Interferon responsive genes are over-represented in the list of genes associated with MS susceptibility. Of the 55 genes identified as associated with MS [2], approximately one-third are identified as interferon responsive in the interferome, the database of interferon-regulated genes (n = 18 genes) [68]. Only one-tenth genes of the 20,000 or so genes now described from the human genome are listed as interferon responsive (n = 1996 genes) [68], so the excess of interferon-responsive genes associated with MS is remarkable (p<E^−8^, Fishers exact test). Our findings raise the possibility that IL7Rα and IFNβ may work together in myeloid cells to affect susceptibility to MS.

## Materials and Methods

The study was approved by the Western Sydney Local Health District Human Research Ethics Committee (HREC2004(1914)) and blood donors gave written informed consent.

### Cell Separation and Culture

Peripheral blood mononucleocytes (PBMCs) were isolated from EDTA-treated whole blood of healthy controls using Ficoll Paque Plus (GE Healthcare, Sweden). The study was approved by the Western Sydney Local Health District Human Research Ethics Committee and blood donors gave informed consent.The age range of donors was 28–67 years, both males and females were included and gender was not significantly different between compared groups. Number of subjects used for each experiment is given in Figure legends. Freshly drawn blood was used for all experiments except Figures 3B, 3C and Suppl Fig 1, where monocytes were purified from thawed cryopreserved PBMCs, Monocytes were isolated by positive CD14+ selection using antibody-conjugated magnetic beads (Miltenyi Biotec, Germany) and the purity obtained was routinely 90%. Immature DCs (iDCs) were prepared from monocytes by incubation with IL-4 (20 ng/ml) and GM.CSF (70 ng/ml) on day 0, 2 and 4 of culture [69]. Maturing DCs (matDC) were induced by the addition of 1 µg/ml LPS on day 5 of culture for 24 h. Plasmacytoid dendritic cells (pDCs) and myeloid dendritic cells (mDCs) were purified as previously described by magnetic separation [70], [71]. The purities of pDC and mDC subsets were routinely 99% and 80% respectively. IFNβ-1a (1000 IU/ml Biogen Idec, Switzerland) was added to freshly purified monocytes, mDCs and to day 5-cultured iDCs (simultaneously with LPS for maturing DC culture) for 24 h. Freshly purified pDCs were cultured with 2000 IU/ml IFNβ-1a for 24 h. All cells were cultured in triplicate at 10^6^/ml at 37°C in 5% CO_2_ in X-Vivo 15 medium (Lonza, Switzerland), washed with phosphate-buffered saline and lysed using Cells-to-Signal Lysis buffer (Ambion, TX, USA). For microarray experiments, fresh monocytes, day 5 iDCs (no LPS) and day 5 maturing DCs (+LPS) were cultured with IFNβ for 48 h before addition of TSLP or IL-7 (10 ng/ml; R&D Systems, MN, USA) for 24 h, followed by washing and lysing as above. For proliferation experiments, and day 5 maturing DCs (+LPS) were cultured with IFNβfor 48 h before addition of TSLP or IL-7 (10 ng/ml; R&D Systems, MN, USA) for 24 h, followed by addition of autologous T cells.

### Genotyping and qRTPCR

Donors were genotyped and IL7Rα haplotype determined as described [13]. For experiments comparing haplotype differences using homozygotes, Hap 1, 2, and 4 were compared, excluding Hap 3 due to lack of availability (low minor allele frequency; [23]). RNA was extracted from cell lysates using the RNeasy Mini RNA extraction Kit (Qiagen, Germany) and reverse-transcribed using Superscript III (Invitrogen, CA, USA) according to the manufacturer's instructions. IL-7Rα semi-quantitative RT-PCR was performed using SYBR Green (Applied Biosystems, CA, USA) with primers spanning intron 7 (Forward: 5′- CTGGAACATCTTTGTAAGAAACCAAG-3′; Reverse: 5′-TAGCTTGAATGTCATCCACCCT-3′). Comparative measurement of mRNA from each haplotype in heterozygotes and determination of the ratio of IL-7Rα mRNA encoding the full length isoform (membrane-bound) to a soluble isoform (exon 6 spliced out) was performed as previously described [13]. Another isoform generated by splicing out of both exon 5 and 6 has recently been described and relative quantitation of this, membrane-bound, and soluble (exon 6 spliced out) IL7Rα was performed as described [41] in a second cohort. Semi-quantification of tolerogenic genes in dendritic cells was performed using SYBR green with primers for IL-27p28 (Forward: 5′-GAGCAGCTCCCTGATGTTTC-3′; Reverse: 5′-AGCTGCAT CCTCTCCATGTT-3′) [72], IDO (Forward: 5′-CAAAGCAGCGTCTTTCAGTG-3′; Reverse: 5′-CGGACTGAGGGATTTG ACTC-3′) and IL-10 (Forward: 5′-TTACCTG GAGGAGGTGATGC-3′; Reverse: 5′-GGCCTTG CTCTTGTTTTCAC-3′).

### Microarray Analysis

Total RNA was extracted from cell lysates using RNeasy Mini RNA extraction Kit (Qiagen, Germany). Total RNA quality was assessed using the Agilent RNA 6000 series II Nano kit (Agilent Technologies, CA, USA), and concentration was measured by NanoPhotometer™ Spectrophotometry (Implen GmbH, Germany). 180 ng total RNA from each sample was biotinylated and amplified using Illumina® TotalPrep RNA Amplification Kit (Ambion, TX, USA). The complementary mRNA (cRNA) yield was then measured using the NanoPhotometer™ Spectrophotometer (Implen GmbH, Germany). 700 ng of cRNA samples were hybridized onto human HT-12_V3 expression beadchips (Illumina, Inc. CA, USA) profiling 48,804 transcripts per sample. Raw data was processed using Beadstudio v3 (Illumina, Inc. CA, USA) which involved filtering of the probe sets to keep only those probes which were detected above background in at least one sample. Data were exported into BRB-ArrayTools version 3.8 and quantile normalization was applied to the data. Genes whose expression levels differed by more than 1.5 fold between groups, were deemed to be differentially expressed. Gene lists were exported into GeneGo™ Metacore (St Joseph, MI, USA) where tests were carried out for over-representation of the gene lists in curated biological pathways. A cutoff of 5% false discovery rate was used to determine significance of over-represented pathways.

### Naïve CD4 T cell Proliferation Assay

CD4+ cells were purified by negative selection, then further purified by positive selection for CD45RA+ cells using antibody-conjugated magnetic beads (Miltenyi Biotec, Germany). Cells were washed twice with PBS and incubated at 10^7^ cells/ml in 10 µM carboxyfluorescein diacetate (CFDA)/PBS for 10 min at 37°C. Cells were washed twice with culture medium and resuspended at a final concentration of 4×10^6^ cells per ml in the DC culture medium. 50 µl of this suspension was added to each well in the presence of anti-CD3/anti-CD28 beads (Invitrogen, CA, USA) (DCs:naïve T cells:beads  = 1∶1∶1). Proliferation was assessed five days later by assessing the reduction in CFDA median fluorescence intensity of CD4+ cells (anti-CD4 by flow cytometry gated on CD4 versus side scatter; LSRII, BD Biosciences, CA, USA).

### Statistics

Comparisons were made between gene expression in the presence and absence of IFNβ, using a paired t test, and between haplotypes or subsets, using the Student's t test assuming unequal variance. Direction of change in response to IFNβ was assessed using the sign test. The comparisons were considered significant if p≤0.05. P values were not corrected for multiple comparisons. Error bars in Figures represent standard error of the mean.

## Supporting Information

Figure S1 **Splicing of IL7Rα is unchanged in MS and upon IFNβ stimulation in myeloid cells of heterozygous Hap 4 carriers.** Cryopreserved PBMCs from healthy controls (n = 5) and MS patients (n = 5) were thawed, monocytes purified, and monocytes (A), *in vitro* cultured immature dendritic cells (IL-4, GM.CSF; iDC) (B) and maturing monocyte-derived dendritic cells (IL-4, GM.CSF, LPS; matDC) (C) were incubated +/− IFNβ (1000 IU/ml) for 24 h. Relative expression of membrane-bound (MB), exon 6 soluble (Sol(-Ex6)), and exon 5,6 soluble (Sol(-Ex5,6)) IL7Rα isoforms was measured as previously described [41] and expressed as a proportion of the total. Mean +/− SEM is shown. There were no significant differences between controls and MS, or between media and IFNβ; bars represent significant differences between isoforms in the same subset under the same condition by paired t test (p<0.05).
(DOCX)
