# Supplementary material for: IL7Rα Expression and Upregulation by IFNβ in Dendritic Cell Subsets Is Haplotype-Dependent
Source: PLoS One. 2013 Oct 16;8(10):e77508. doi: 10.1371/journal.pone.0077508 (PMC3797747; doi:10.1371/journal.pone.0077508)
Supplement: Figure S1 — Splicing of IL7Rα is unchanged in MS and upon IFNβ stimulation in myeloid cells of heterozygous Hap 4 carriers. Cryopreserved PBMCs from healthy controls (n = 5) and MS patients (n = 5) were thawed, monocytes purified, and monocytes (A), in vitro cultured immature dendritic cells (IL-4, GM.CSF; iDC) (B) and maturing monocyte-derived dendritic cells (IL-4, GM.CSF, LPS; matDC) (C) were incubated +/− IFNβ (1000 IU/ml) for 24 h. Relative expression of membrane-bound (MB), exon 6 soluble (Sol(-Ex6)), and exon 5,6 soluble (Sol(-Ex5,6)) IL7Rα isoforms was measured as previously described [41] and expressed as a proportion of the total. Mean +/− SEM is shown. There were no significant differences between controls and MS, or between media and IFNβ; bars represent significant differences between isoforms in the same subset under the same condition by paired t test (p<0.05). (DOCX) [file pone.0077508.s001.docx]

|  | CONTROL | MS |
| --- | --- | --- |
| mono | **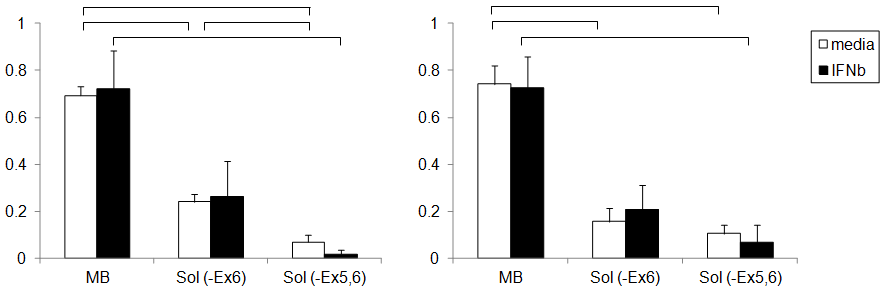** | |
| iDC | **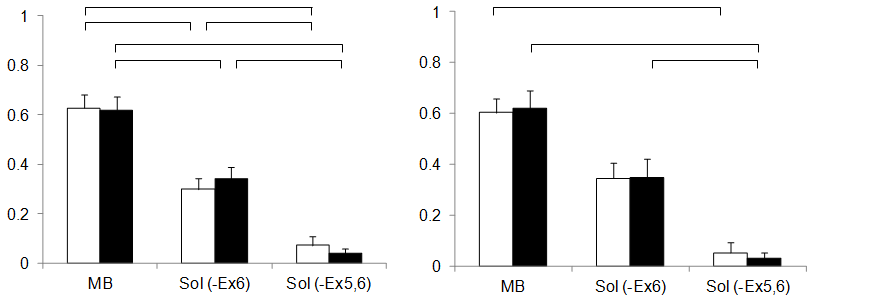** | |
| matDC | **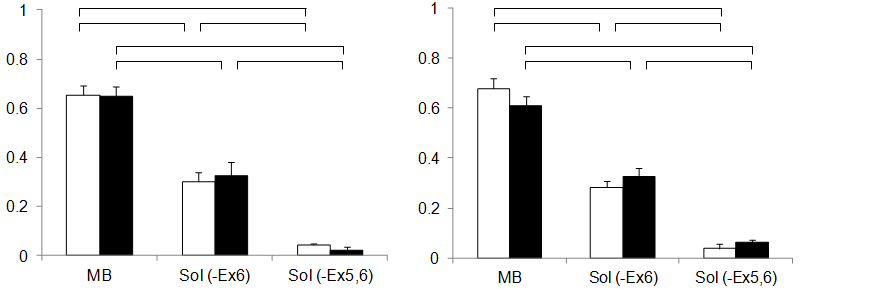** | |

**SUPPORTING FIGURE 1** Splicing of IL7Rα is unchanged in MS and upon IFNβ stimulation in myeloid cells of heterozygous Hap 4 carriers. Cryopreserved PBMCs from healthy controls (n = 5) and MS patients (n = 5) were thawed, monocytes purified, and monocytes (A), *in vitro* cultured immature dendritic cells (IL-4, GM.CSF; iDC) (B) and maturing monocyte-derived dendritic cells (IL-4, GM.CSF, LPS; matDC) (C) were incubated +/- IFNβ (1000 IU/ml) for 24 h. Relative expression of membrane-bound (MB), exon 6 soluble (Sol(-Ex6)), and exon 5,6 soluble (Sol(-Ex5,6)) IL7Rα isoforms was measured as previously described [[41](#_ENREF_41)] and expressed as a proportion of the total. Mean +/- SEM is shown. There were no significant differences between controls and MS, or between media and IFNβ; bars represent significant differences between isoforms in the same subset under the same condition by paired t test (p < 0.05).
